# Synthesis and Anti-Inflammatory Activity of 1-Methylhydantoin Cinnamoyl Imides

**DOI:** 10.3390/molecules27238481

**Published:** 2022-12-02

**Authors:** Shihan Wang, Li Ji, Dongxue Zhang, Hongye Guo, Yongsheng Wang, Wei Li

**Affiliations:** 1 College of Chinese Medicinal Materials, Jilin Agricultural University, Changchun 130118, China; 2 School of Pharmaceutical Sciences, Jilin University, Changchun 130021, China

**Keywords:** *Oviductus Ranae*, natural product, inflammatory, 1-Methylhydantoin, *trans*-cinnamic acid

## Abstract

In this study, 1-methylhydantoin cinnamic imides were synthesized from 1-methylhydantoin and *trans*-cinnamic acid, and their anti-inflammatory activity was investigated. The anti-inflammatory activity in vitro was evaluated by measuring the contents of NO, TNF-α and IL-1β in the supernatant of RAW264.7 cells stimulated by LPS. The cytotoxicity of 1-methylhydantoin cinnamoyl imides on RAW264.7 cells was detected using the CCK-8 method. The results showed that compounds **2** and **4** can significantly inhibit the release of NO and reduce the secretion of TNF-α and IL-1β. Compound **3** inhibited the production of TNF-α. The inhibition rate of COX was evaluated in vitro. The in vivo anti-inflammatory activities of the five compounds were evaluated by establishing an animal model of xylene ear swelling. The results showed that 1-methylhydantoin cinnamic imides could alleviate xylene-induced ear edema in mice in a dose-dependent manner. Among them, the effect of compound **5** was the most significant. Under the action of high dosage, its ear swelling inhibition rate was as high as 52.08%.

## 1. Introduction

Inflammation is the body’s natural and necessary response to stimuli. It promotes the production of cytokines, chemokines and acute-phase proteins, and coordinates a series of steps such as transport, enabling immune system cells to migrate toward stimulation targets [1]. On the one hand, a quick response to inflammation can protect the body, remove harmful stimuli and gradually restore the injured area to its original state. On the other hand, excessive and chronic inflammation can lead to tissue damage and fibrosis. Therefore, it is closely related to various diseases, such as joint diseases [2], gastrointestinal diseases [3], cardiovascular diseases [4], neurodegenerative diseases and various diffuse diseases [5,6]. At present, the toxic side effects of non-steroidal anti-inflammatory drugs (NSAIDs) have seriously endangered human health, so the development of new anti-inflammatory drugs is of great significance. Natural products have the characteristics of high safety and strong biological activity [7]. The development of new drugs based on natural products has gained broad consensus [8,9,10].

**Figure 1:**
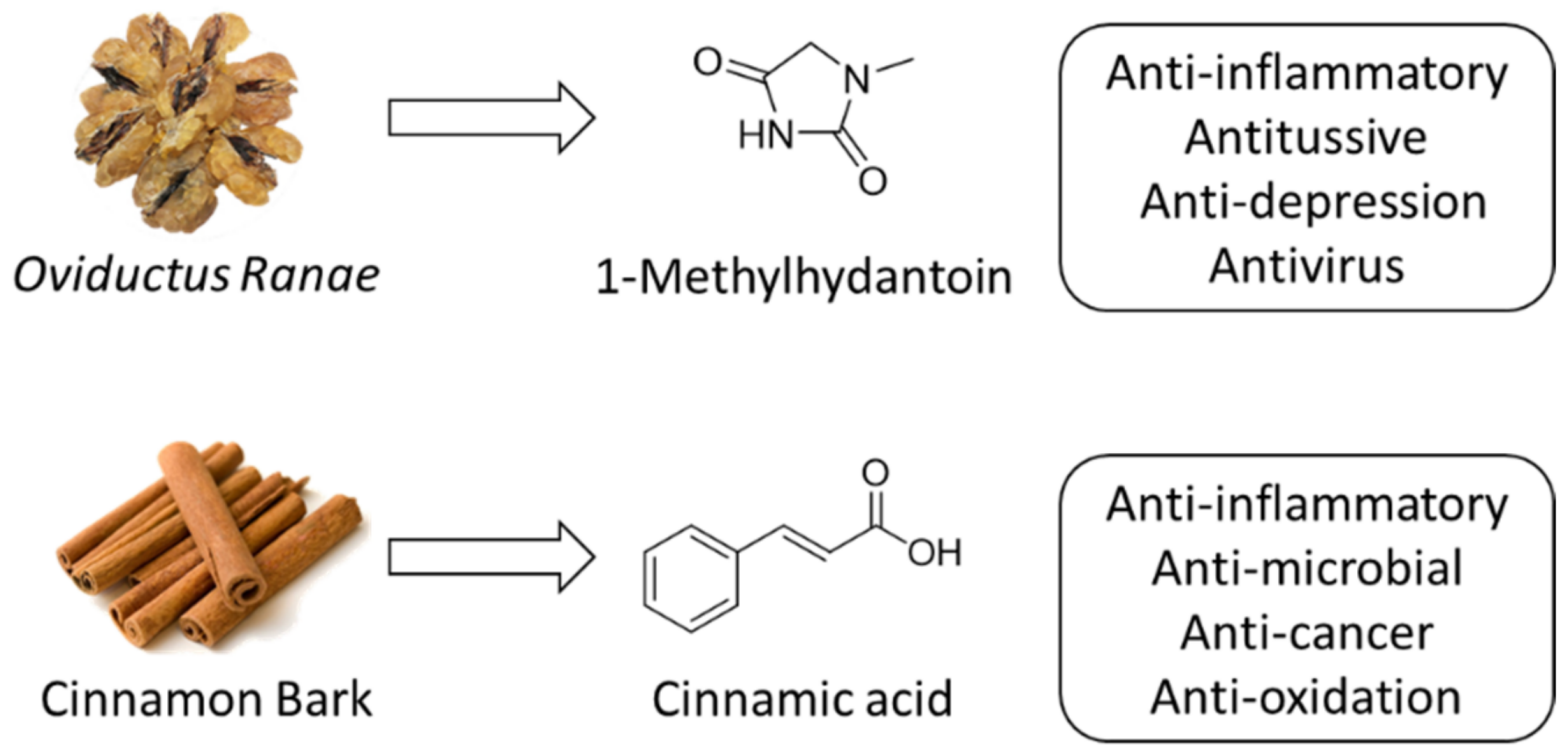
The 1-methylhydantoin was isolated from a traditional Chinese medicine, *Oviductus Ranae*. *Trans*-Cinnamic acid is a natural product that can be isolated from cinnamon bark.

1-Methylhydantoin (MHD) is a natural product that was isolated from traditional Chinese medicine, *Oviductus Ranae* (Chinese Pharmacopoeia and Figure 1). 1-Mehylhydantoin was reported to have various bioactivates, such as antivirus and antitussive [11,12]. In our previous study, it was found that the natural product 1-methylhydantoin can reduce ear edema in mice caused by xylene, showing good anti-inflammatory activity [13,14]. On the other hand, *trans*-cinnamic acid is a natural organic acid compound that exists in a variety of plants, such as cinnamon bark (Figure 1), in a single form or in complex structures [15]. It is commonly used in spices, food additives and pharmaceutical intermediates [16]. Studies have shown that *trans*-cinnamic acid and its natural and synthetic derivatives have a variety of biological effects, such as anti-microbial [17], anti-cancer [18], anti-oxidation and anti-inflammatory [17,19]. Using the principle of drug splicing to modify the structure of 1-methylhydantoin has the possibility of improving anti-inflammatory activity. Existing studies have shown that cinnamamide compounds have several potential active sites, such as styryl, amide and aryl groups. Compounds with these active sites are more effective than simple aromatic amides [20]. The related literature also confirms that cinnamamide derivatives have a variety of biological activities, such as anti-cancer activity [21], antibacterial activity [22], anti-tuberculosis activity [23], anticonvulsant activity [24] and anti-inflammatory activity [25,26,27]. There are a large number of patented drugs that also have the structure of cinnamamide, including patented anti-cancer drugs, patented anti-lipid drugs and patented drugs for the treatment of respiratory diseases [28]. Cinnamamide has great biological properties. When amine compounds with intrinsic activity are selected and the principle of drug combination and *trans*-cinnamic acid compounds are used to convert them into cinnamamide, it is more likely to be converted into effective compounds and further enhance the activity. 

In our previous work, the anti-inflammatory effect of 1-methylhydantoin has been demonstrated. Meanwhile, 1-methylhydantoin exhibited a great binding affect to COX through hydrogen bonding and van der Waals force. A molecular docking study showed the potential to improve its anti-inflammatory effect when linked to cinnamic groups. Herein, this work is dedicated to optimizing 1-methylhydantoin and exploring the potential targets using cinnamoyl moieties. 1-Methylhydantoin cinnamic imides were prepared and their purity was determined through the HPLC-UV method. Lipopolysaccharide (LPS)-induced RAW264.7 cells were used to evaluate the cytotoxicity and preliminary anti-inflammation effects. The in vivo experiments were conducted based on the xylene-induced ear inflammation model.

## 2. Results and Discussion

**Scheme 1:**
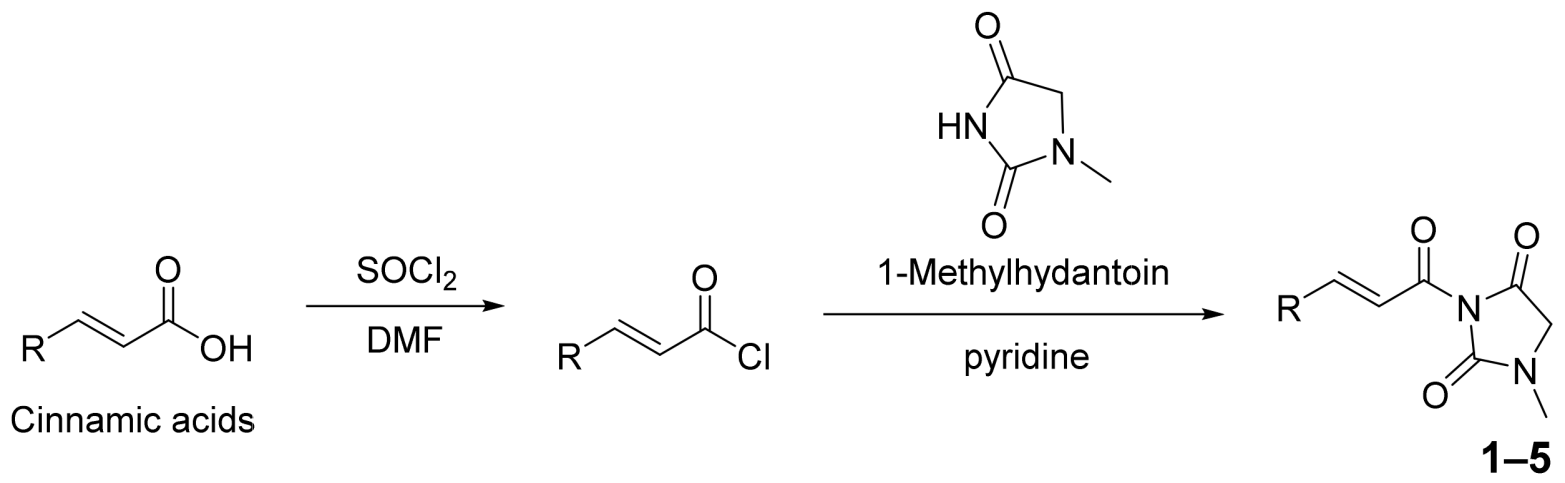
The synthetic approach of 1-methylhydantoin cinnamic imides.

**Figure 2:**
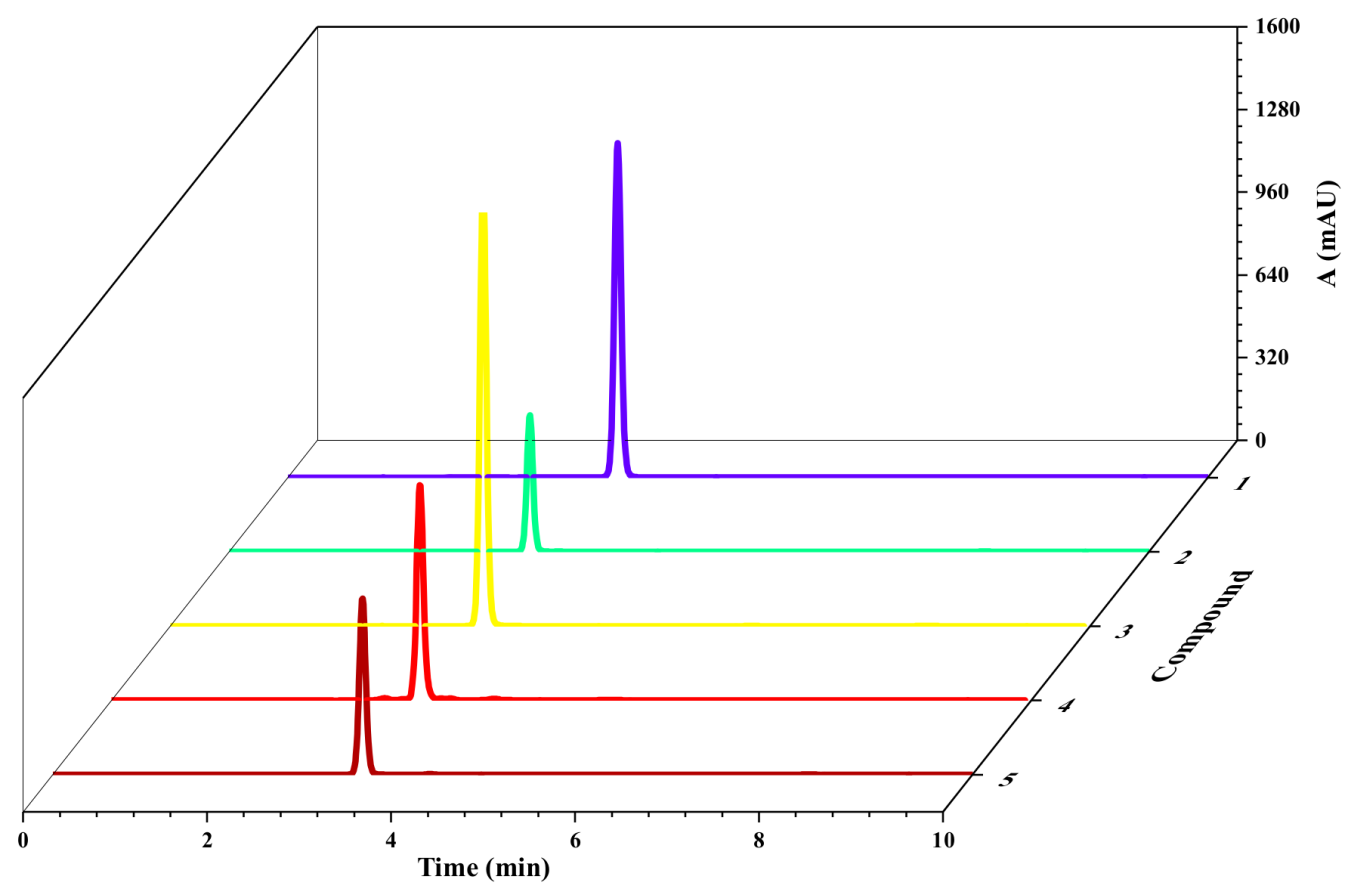
The purity of synthetic 1-methylhydantoin cinnamic imides was determined using HPLC-UV chromatogram at 254 nm.

The target compounds (Scheme 1) were mainly prepared through acid chloride reaction and amide reaction. In short, SOCl_2_ was used to activate the carboxyl group of the *trans*-cinnamic acid compound and carry out a nucleophilic substitution reaction with the amine group of 1-methylhydantoin. The target compounds (**1**–**5**) were separated and purified using column chromatography and recrystallization. The yields of the target compounds (**1**–**5**) were 37% to 61%. The purity of 1-methylhydantoin cinnamic imides was determined using High Performance Liquid Chromatography (HPLC). The chromatogram is shown in Figure 2. According to the chromatogram and chromatographic peak information (Table 1), it can be seen that the five target compounds are at 254 nm the detection wavelength, the peak number is single and there was no obvious impurity. The peak areas of the five compounds account for 99.7%, 99.0%, 99.7%, 95.5% and 98.8%, respectively, which indicates that the five target compounds have high purity and are subsequent activities.

The inhibition ability of 1-methylhydantoin cinnamic imides to ovine cyclooxygenase-1 (COX-1) and cyclooxygenase-2 (COX-2) was determined using an enzyme immunoassay kit. The results showed that five compounds have IC50 of COX-1 in a range of 37 ± 4 to 56 ± 6 μM and IC50 of COX-2 in a range of 126 ± 12 to 204 ± 10 μM. Compound **4** showed the best inhibition ability with IC50 COX-1 37 ± 4 μM and COX-2 126 ± 12 μM, while compound **2** showed IC50 COX-1 56 ± 6 μM and COX-2 204 ± 10 μM. The Cell Counting Kit-8 (CCK-8) method was used to detect the cytotoxicity of the five target compounds (**1**–**5**) on lipopolysaccharide (LPS)-induced RAW264.7 cells. The results of half maximal cytotoxic concentration (CC50) and 95% survival safe conventions are shown in Table 2. When the concentrations of compounds **1**, **3** and **5** are lower than 20 μM, and when the concentrations of compounds **2** and **4** are lower than 80 μM, the survival rate of RAW264.7 cells is more than 95%, which has no obvious damage to cell survival and can be used for subsequent in vitro anti-inflammatory activity experiments to provide the basis.

**Figure 3:**
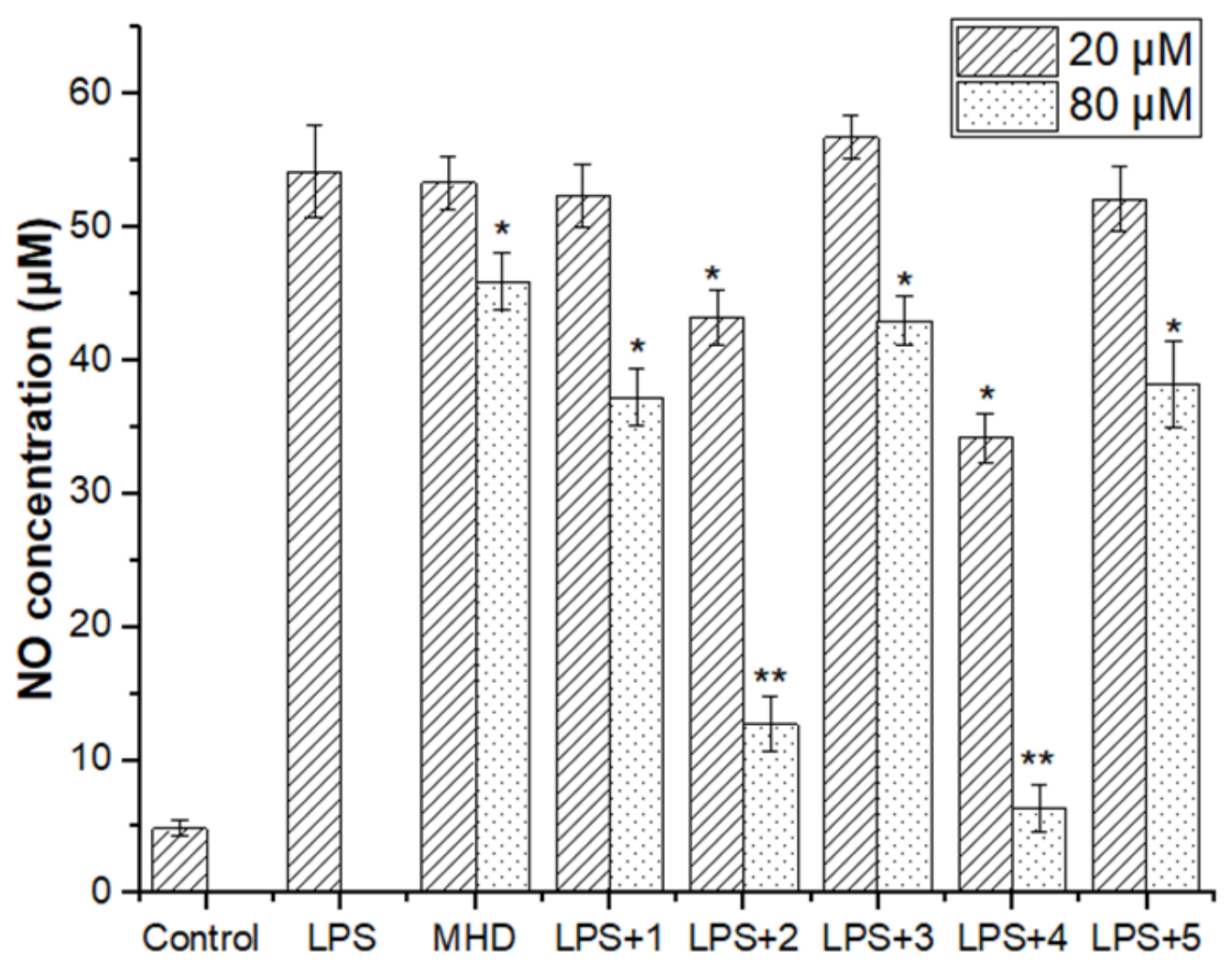
The effect of 1-methylhydantoin cinnamic imides on the release of NO from RAW264.7 cells induced by LPS. (n = 3, * *p* < 0.05, ** *p* < 0.01 vs. the LPS-treated group).

NO is an important indicator to predict the anti-inflammatory effects of drugs. It has been identified as a proinflammatory cytokine in various inflammatory diseases. NO can induce vasodilation, recruit neutrophils and inhibit the activation of mast cells, leading to tissue damage. It is essential to reduce the production of NO for inflammatory release [29,30]. To explore the anti-inflammatory effects of the five compounds, the LPS-induced inflammatory response of RAW264.7 cells was carried out. The NO content in the cell supernatant was measured by the Griess method to evaluate the inhibitory effects of these five compounds on NO production. It can be seen from Figure 3 that the LPS-induced inflammation model group and each experimental group had significantly higher NO content than the blank group. Among them, the inhibitory rate of NO on compounds **1-5** (20 μM) was not obvious, while compounds **2** and **4** (80 μM) showed a significant effect on NO. Compared with the MHD, they obviously inhibit the production of NO and have obvious anti-inflammatory effects.

**Figure 4:**
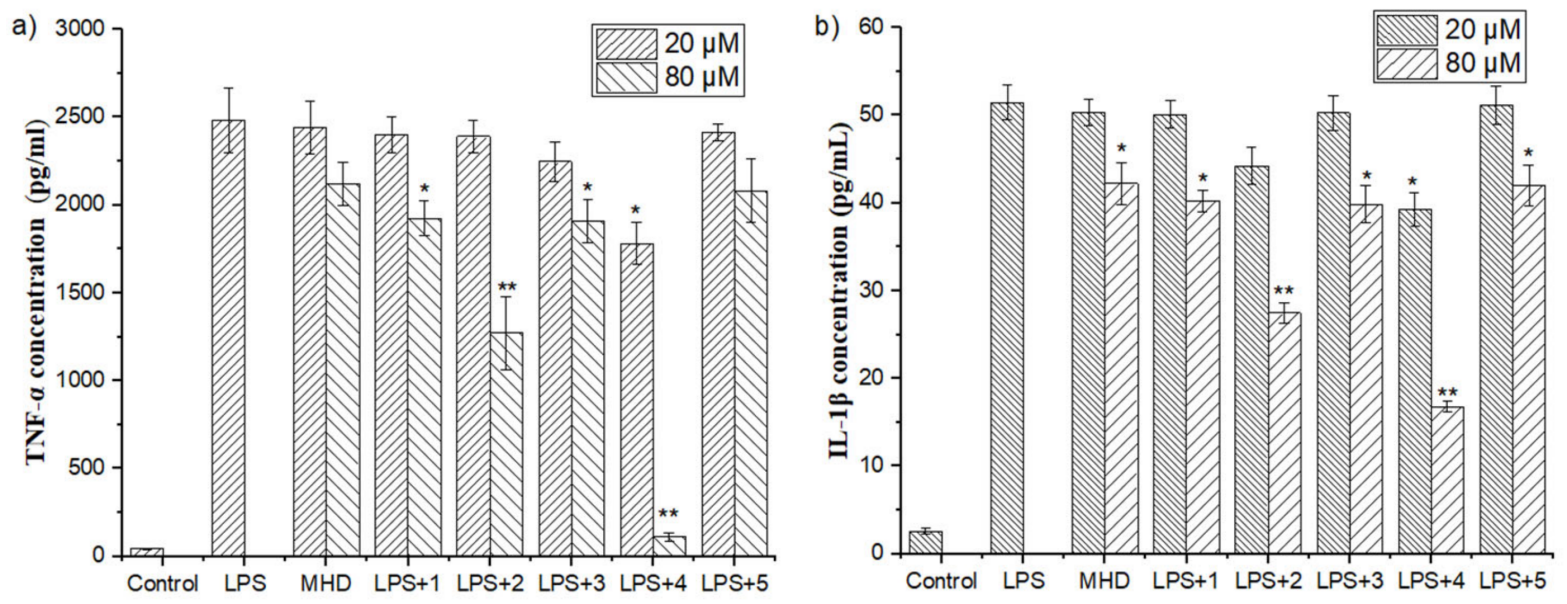
(**a**) The effect of 1-methylhydantoin cinnamic imides on the secretion of TNF-α in RAW264.7 cells induced by LPS. (**b**) The effect of 1-methylhydantoin cinnamic imides on the secretion of IL-1β in RAW264.7 cells induced by LPS. (n = 3, * *p* < 0.05, ** *p* < 0.01 vs. the LPS-treated group).

Tumor necrosis factor-α (TNF-α) is a pleiotropic proinflammatory cytokine, known for its proinflammatory activity [31]. To explore the inhibitory effects of five compounds on TNF-α, ELISA was used to determine the content of inflammatory factor TNF-α in the supernatant of RAW264.7 cells stimulated by LPS. RAW264.7 cells secreted a large amount of inflammatory-factor TNF-α 24 h after LPS (1 ug/mL) induction. Among them, compounds **1**, **2**, **3** and **5** (20 μM) had no significant difference between the experimental group and the model group and had no obvious inhibitory effect on TNF-α induced by LPS. Compared with the model group, the compounds **2** and **4** (80 μM) in the experimental group were significantly different and could significantly reduce the secretion of TNF-α (Figure 4a). Among them, the inhibitory effect of compound **4** is the most significant, and the TNF-α content was similar to the control group.

Interleukin-1β (IL-1β) has strong proinflammatory activity and can induce the production of a variety of proinflammatory mediators, such as cytokines and chemokines [32,33]. In this work, the ELISA method was used to determine the IL-1β content in the supernatant of RAW264.7 cells stimulated by LPS to evaluate the effects of five compounds on IL-1β secretion. The results are shown in Figure 4b. LPS-induced RAW264.7 cells were shown to secrete a large amount of IL-1β after 24 h, and the IL-1β content of the model group was significantly higher than that of the blank group. Compounds **1**, **2**, **3** and **5** (20 μM) in the experimental group were not significantly different from the model group and had no obvious inhibitory effect on IL-1β induced by LPS, while when compounds **2** and **4** (80 μM) are compared with the model group, the difference is significant and they have a significant inhibitory effect on the secretion of IL-1β.

**Figure 5:**
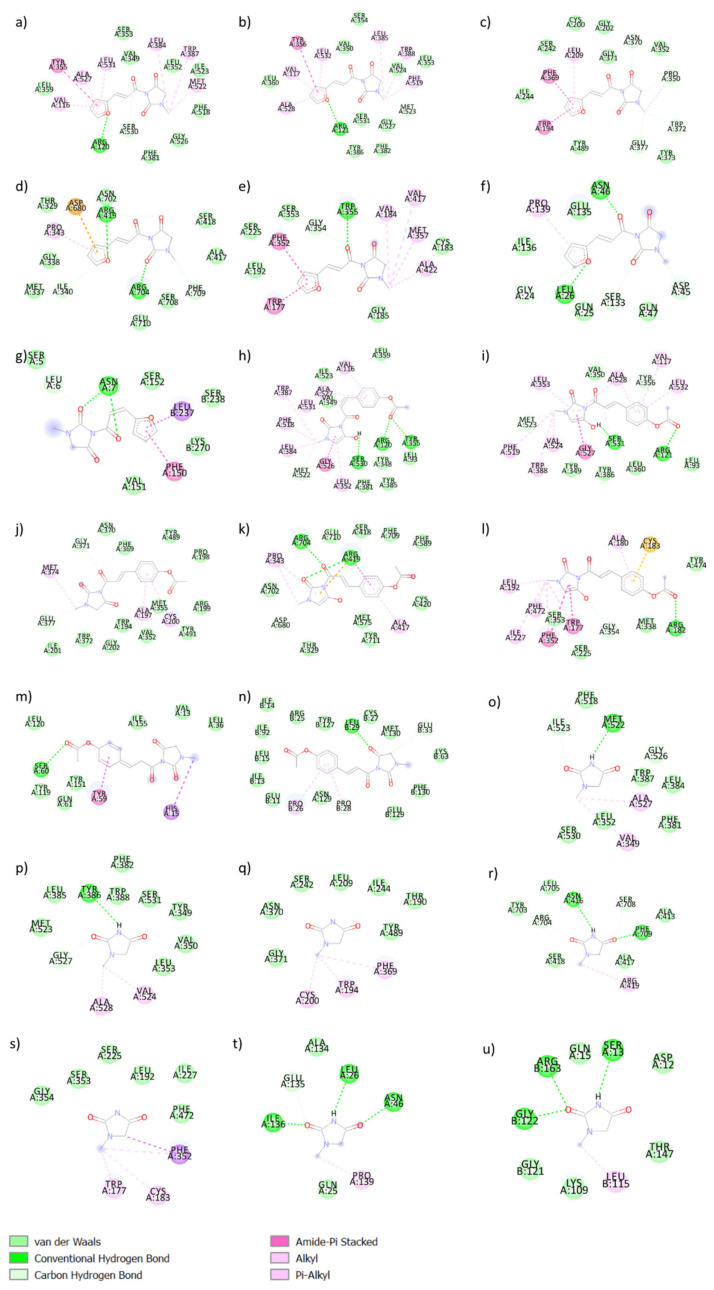
Docking results of compound **2** with COX-1 (**a**), COX-2 (**b**), iNOS (**c**), nNOS (**d**), eNOS (**e**), TNF-α (**f**) and IL-1β (**g**); compound **4** with COX-1 (**h**), COX-2 (**i**), iNOS (**j**), nNOS (**k**), eNOS (**l**), TNF-α (**m**) and IL-1β (**n**); and MHD with COX-1 (**o**), COX-2 (**p**), iNOS (**q**), nNOS (**r**), eNOS (**s**), TNF-α (**t**) and IL-1β (**u**).

The binding affinity of a ligand to a target is indispensable for tight association, to attenuate the drug concentration and to lower the risk of side effects engendered by nonspecific or nontarget binding during treatment. The molecular docking study aimed to explore the possible interactions of five target compounds with the crystal structure of COX-1, COX-2, NOS, TNF-α and IL-1β. The docking scores (kcal/mol) of potential active compounds (**1**–**5**) to three target proteins are shown in Table 3. Compounds **1**–**5** have remarkable binding affinity with COX, NOS and IL-1β compared with MHD. The binding with TNF-α is weaker compared to the other two proteins. The binding models of compounds **2** and **4** with COX, NOS, TNF-α and IL-1β are shown in Figure 5. Compounds **1**–**5** showed greater binding affinities of NOS than COX, TNF-α and IL-1β (Table 3). Compound **2** showed two hydrogen bonds with nNOS ARG419A and ARG704A, one hydrogen bond with TNF-α ASN46A (2.085 Å) and three hydrogen bonds with IL-1β ASN7A (1.960, 2.464, 3.060 Å) (Figure 5a–e). Compound **4** showed one hydrogen bond with IL-1β LEU29B (2.304 Å) (Figure 5f–i).

**Figure 6:**
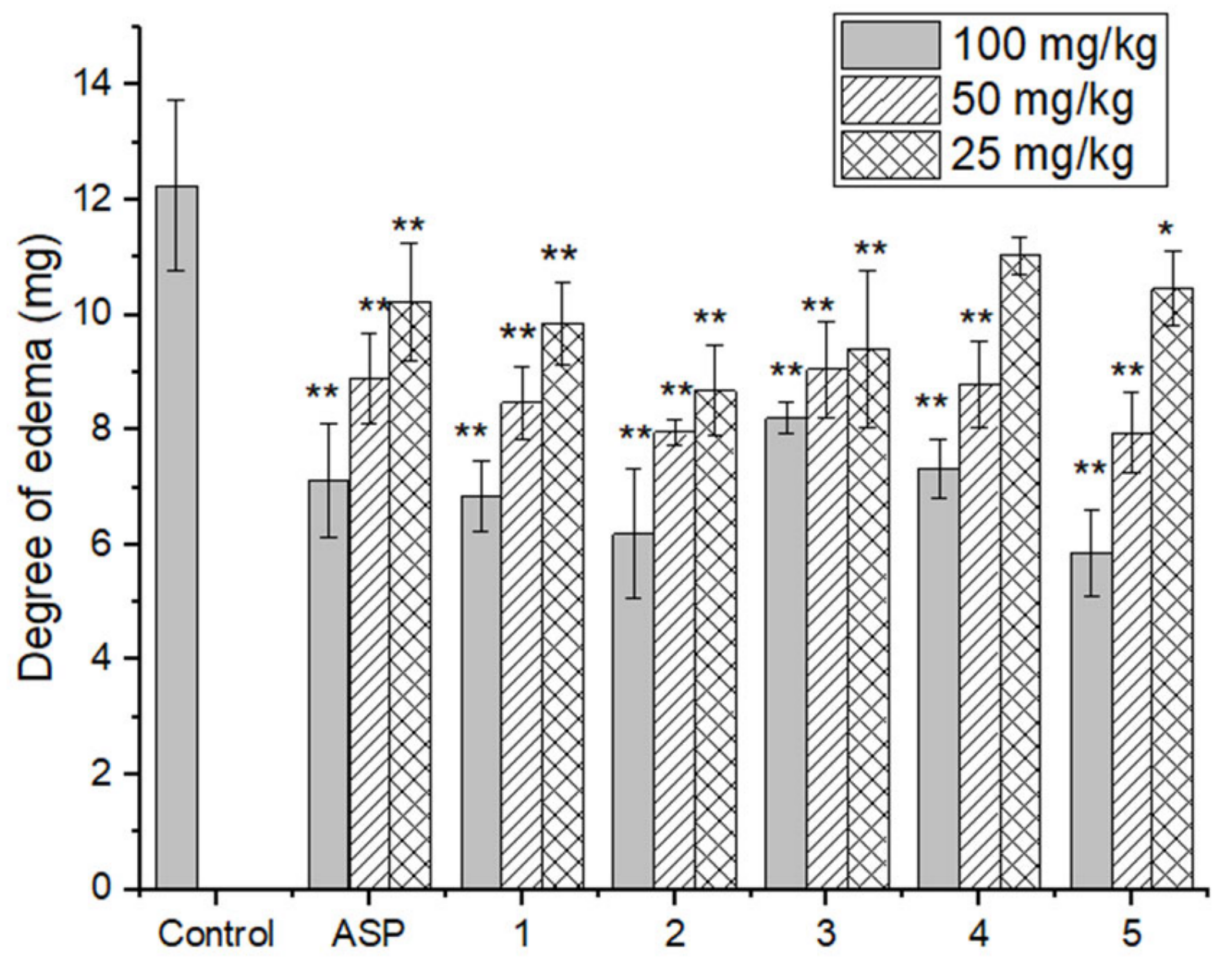
The bioactivity of 1-methylhydantoin cinnamic imides on the swelling degree of xylene-induced ear edema in mice with aspirin as the positive control. The experiment was carried out using high, medium, and low dosages of five compounds. (n = 6, * *p* < 0.05, ** *p* < 0.01 vs. control).

To evaluate the differences in anti-inflammatory effects of the five compounds, the in vivo anti-inflammatory experiment was conducted based on the acute inflammation model of ear swelling induced by xylene. Under the inflammatory effect of 30 μL of xylene, the degree of ear swelling in the blank group and each experimental group was different. The degree of ear swelling in the experimental group was significantly lower than that in the blank group and the inhibition rates of ear swelling in each experimental group were significantly different, indicating that the five target compounds have different degrees of anti-inflammatory effect. Table 4 shows that the anti-inflammatory effects of the five compounds are dose dependent. With an increase in dose the degree of ear swelling decreased, and the effect of ear swelling inhibition was more significant at high dose (100 mg/kg). Under the action of drugs, the five target compounds (**1–5**) have significant inhibitory effects on ear swelling (Figure 6). Among them, compound **5** has the most obvious anti-inflammatory effect in vivo, with an inhibitory rate as high as 52.08%.

## 3. Materials and Methods

### 3.1. Materials

Target compounds (**1**–**5**) were synthesized in our laboratory. The purity of target compounds (**1**–**5**) was ≥ 95% using HPLC analysis (Agilent Technologies 1260, Pittsburgh, PA, USA). HPLC-grade methanol and acetonitrile were purchased from Xinkeao Scientific and Technology Co., Ltd. (Beijing, China). CCK-8 and Griess kits were purchased from Promega Corporation (Madison, WI, USA). TNF-α and IL-1β ELISA kits were purchased from Proteintech (Rosemont, IL, USA). The COX enzyme immunoassay kit was purchased from Cayman Chemical (Ann Arbor, MI, USA). Other chemicals and reagents were of analytical grade and commercially available. All chemicals and reagents were used without further purification unless otherwise stated. The reaction was monitored using thin layer chromatography (TLC) and a silica-gel-coated glass plate (Qingdao Ocean Chemical Co., Qingdao, China), and visualized under UV light (254 nm). An FTIR spectrum was carried out on IRPrestige-21, SHIMADZU. MS was recorded using Q-TOF SYNAPT G2 HDMS, Waters. ^1^H-NMR and ^13^C-NMR were recorded on a Bruker AVANCE 500 NMR spectrometer (Fällanden, Switzerland) and chemical shifts were reported in ppm. Melting point was recorded on an SGW^®^X-4 microscope melting apparatus (Shanghai Scientific and Technology Co., Ltd., Shanghai, China). NMR, FTIR, UV/Vis and MS spectra of target compounds are given in Supplementary Materials.

### 3.2. General Synthetic Method

Cinnamic acid (0.50 g, 3.4 mmol) was dispersed evenly in 10 mL CH_2_Cl_2_.SOCl_2_ (0.28 mL, 3.74 mmol), 1 drop of catalyst N,N-dimethylformamide (DMF) was added to the solution and the solution was stirred at room temperature for 6 h. 1-methylhydantoin (0.35 g, 3.06 mmol) was then suspended in 10 mL CH_2_Cl_2_ with pyridine (0.6 mL, 7.48 mmol). It was placed in ice-water at 0 °C and stirred for 10 min. Cinnamic acid chloride solution was placed in an ice bath and added dropwise and slowly to the 1-methylhydantoin CH_2_Cl_2_ solution. After stirring at room temperature for 6 h, pyridine was removed, and the reaction was purified using column chromatography. Petroleum ether/ethyl acetate (1:1.5) was eluted under reduced pressure to obtain compound **1**.

### 3.3. 3-cinnamoyl-1-methylimidazolidine-2,4-dione (***1***)

A white solid, with a yield of 56%. Mp: 140.6–141.5 ℃. MS [C_13_H_12_N_2_O_3_ + Na]^+^ 267.0247. IR (cm^−1^): 3107, 3069, 2939, 1799, 1732, 1612, 1323, 1279, 1150, 935, 764. ^1^H-NMR (400 MHz, CDCl_3_) δ 7.93 (d, *J* = 15.7 Hz, 1H), 7.65–7.58 (m, 2H), 7.47–7.33 (m, 4H), 3.98 (s, 2H), 3.06 (s, 3H). ^13^C-NMR (100 MHz, CDCl_3_) δ 167.3, 163.1, 153.3, 147.9, 134.2, 131.1, 128.9, 128.82, 118.8, 77.3, 77.0, 76.7, 51.3, 29.7.

### 3.4. (E)-3-(3-(furan-2-yl)acryloyl)-1-methylimidazolidine-2,4-dione (***2***)

A beige solid, with a yield of 59%. Mp: 128.9–129.8 ℃. MS [C_11_H_10_N_2_O_4_ + Na]^+^ 257.0050. IR (cm^−1^): 3107, 2359, 2332, 1803, 1744, 1610, 1472, 1329, 1277, 970, 849. ^1^H-NMR (400 MHz, CDCl_3_) δ 7.66 (d, *J* = 15.4 Hz, 1H), 7.53 (s, 1H), 7.19 (d, *J* = 15.3 Hz, 1H), 6.76 (s, 1H), 6.51 (s, 1H), 3.98 (s, 2H), 3.05 (s, 3H). ^13^C-NMR (100 MHz, CDCl_3_) δ 167.3, 163.1, 153.3, 151.1, 145.7, 133.5, 117.3, 116.3, 112.8, 77.4, 77.0, 76.7, 51.5, 29.6.

### 3.5. (E)-3-(3-(benzo[d][1,3]dioxol-5-yl)acryloyl)-1-methylimidazolidine-2,4-dione (***3***)

A white solid, with a yield of 61%. Mp: 164.8–166.1 ℃. MS [C_14_H_12_N_2_O_5_ + Na]^+^ 311.0255. IR (cm^−1^): 2361, 1790, 1614, 1599, 1450, 1288, 1258, 1041, 937, 814, 748. ^1^H-NMR (400 MHz, CDCl_3_) δ 7.83 (d, *J* = 15.6 Hz, 1H), 7.17 (d, *J* = 15.5 Hz, 1H), 7.13–7.07 (m, 2H), 6.82 (d, *J* = 7.9 Hz, 1H), 6.02 (s, 2H), 3.97 (s, 2H), 3.05 (s, 3H). ^13^C-NMR (101 MHz, CDCl_3_) δ 167.3, 163.1, 153.4, 150.5, 148.4, 147.8, 128.7, 125.8, 116.7, 108.6, 106.9, 101.7, 77.3, 77.0, 76.7, 51.2, 29.6.

### 3.6. (E)-4-(3-(3-methyl-2,5-dioxoimidazolidin-1-yl)-3-oxoprop-1-en-1-yl)phenyl acetate (***4***)

A white solid, with a yield of 37%. Mp: 148.9–149.6 ℃. MS [C_15_H_14_N_2_O_5_ + Na]^+^ 325.0409. IR (cm^−1^): 2366, 1794, 1610, 1508, 1400, 1329, 1225, 1159, 982, 912, 845. ^1^H-NMR (400 MHz, CDCl_3_) δ 7.90 (dd, *J* = 15.7, 3.7 Hz, 1H), 7.63 (dd, *J* = 8.9, 2.4 Hz, 2H), 7.38–7.29 (m, 1H), 7.18–7.11 (m, 2H), 3.98 (d, *J* = 1.5 Hz, 2H), 3.05 (d, *J* = 2.6 Hz, 3H), 2.31 (d, *J* = 1.8 Hz, 3H). ^13^C-NMR (100 MHz, CDCl_3_) δ 169.0, 167.2, 162.9, 153.3, 152.7, 146.7, 131.9, 130.0, 122.2, 118.9, 77.3, 77.0, 76.7, 51.2, 29.6, 21.1.

### 3.7. (E)-2-methoxy-4-(3-(3-methyl-2,5-dioxoimidazolidin-1-yl)-3-oxoprop-1-en-1-yl)phenyl acetate (***5***)

A white solid, with a yield of 42%. Mp: 148.3–149.8 ℃. MS [C_16_H_16_N_2_O_6_ + Na]^+^ 355.0541. IR (cm^−1^): 2359, 1769, 1618, 1506, 1420, 1335, 1263, 1182, 1128, 905, 843. ^1^H-NMR (400 MHz, CDCl_3_) δ 7.88 (d, *J* = 15.6 Hz, 1H), 7.32 (dd, *J* = 15.6, 1.3 Hz, 1H), 7.22 (d, *J* = 8.2, 1.6 Hz, 1H), 7.16 (d, *J* = 1.6 Hz, 1H), 7.07 (dd, *J* = 8.2, 1.3 Hz, 1H), 3.98 (d, *J* = 1.4 Hz, 2H), 3.88 (d, *J* = 1.3 Hz, 3H), 3.06 (d, *J* = 1.4 Hz, 3H), 2.32 (d, *J* = 1.4 Hz, 3H). ^13^C-NMR (100 MHz, CDCl_3_) δ 168.64, 167.2, 162.9, 153.2, 151.4, 147.1, 142.1, 133.1, 123.3, 121.9, 119.0, 112.0, 77.3, 77.0, 76.7, 56.0, 51.2, 29.6, 20.6.

### 3.8. In Vitro COX Inhibition Assay

The inhibition ability of ovine COX*-*1 and COX*-*2 was determined using an enzyme immunoassay kit (560131, Cayman Chemical, Ann Arbor, MI, USA) following the manufacturer’s instructions.

### 3.9. Cell Culture

RAW264.7 mouse macrophages were purchased from Zhongqiao Xinzhou and cultured in DMEM supplemented with 10% fetal bovine serum (FBS) and 1% penicillin/streptomycin in a 37 °C, 5% CO_2_ incubator. The cells were passaged every two days to maintain the monolayer cell state.

### 3.10. Cytotoxicity Determination

The CCK-8 kit was used to determine the cytotoxicity of the target compounds (**1**–**5**) to RAW264.7 cells. The well-growing cells were subcultured in a 96-well plate. After the cells adhered to the wall, the experimental group was added with a medium containing different concentrations of compounds to make the final concentration reach 5, 10, 20, 40, 80, 160 and 320 μmoL/L. The control group was added with a medium without compound, and a blank group without cells or compound was set at the same time. After culturing in a constant temperature incubator for a period, CCK-8 solution was added to produce yellow formazan product. The OD value was measured by the microplate reader at the detection wavelength of 450 nm, and the cell survival rate was calculated. The cell survival rate formula is as follows:

(1) $$\mathrm{Cell}\;\mathrm{Survival}\;\mathrm{Rate}\;\left(\%\right)=\left[\frac{{\mathrm{OD}}_{e}-{\mathrm{OD}}_{b}}{{\mathrm{OD}}_{c}-{\mathrm{OD}}_{b}}\right]\times 100\%$$

where ${\mathrm{OD}}_{e}$ was the OD value of the experimental group, ${\mathrm{OD}}_{b}$ was the OD value of the blank group, and ${\mathrm{OD}}_{c}$ was the OD value of the control group.

### 3.11. Determination of NO, TNF-α, IL-1β Content

The RAW264.7 cells in the logarithmic growth phase were seeded in a 24-well plate and placed in a 37 °C, 5% CO_2_ constant temperature incubator for culture. After 24 h, the culture medium was discarded. The blank group was added with 10% FBS DMEM 1 mL. The group was added with a medium containing 1 ug/mL LPS, and the experimental group was added with LPS and different concentrations of the target compound to make the final volume 1 mL. After 24 h of culture, the cell supernatant was taken and stored in a refrigerator at −20 °C for later use. The Griess kit was used to determine the concentration of NO in the supernatant, and a double antibody sandwich ELISA kit was used to detect TNF-α and IL-1β.

### 3.12. Molecular Docking

The binding affinity was evaluated using MGLTools of AutoDock 4.2. The crystal structure of COX-1 (PDB ID: 3N8Z), COX-2 (4PH9), iNOS (PDB ID: 4CX7), nNOS (PDB ID: 4UCH), eNOS (PDB ID: 3E7S), TNF-α (PDB ID: 2AZ5) [34] and IL-1β (PDB ID: 1ITB) [35] were retrieved from Protein Data Bank. Polar hydrogen atoms were added and Gasteiger charge was applied. The conformation with the highest binding affinity and reasonable ability was selected for further analysis.

### 3.13. Animals

Adult male Kunming mice (18–20 g) were bought from Liaoning Changsheng Biotechnology Co., Ltd. (Shenyang, China, License No. SCXK (Liao) 2015-0001). The breeding is carried out under standard conditions without pathogens (constant room temperature 20–22 °C, 55 ± 5% humidity, 12 h light–dark cycle and free access to food and water). The research protocol has been approved by the Animal Experiment Ethics Committee of the School of Pharmacy of Jilin University (20190048). All experimental operations follow the principles of laboratory animal care, and all animal experiments are conducted in accordance with the Guide for the Care and Use of Laboratory Animals (released by the National Research Council).

### 3.14. Anti-Inflammatory Activity In Vivo

In this experiment, Kunming mice were randomly divided into two groups. One group was given 5% sodium carboxymethyl cellulose solution as the blank group, and the other group was given different concentrations of the target compound as the experimental group (high, medium and low doses); continuous intragastric administration was two days and the volume of intragastric administration was 0.2 mL. One hour after the last administration, 30 μL of xylene was evenly smeared on both sides of the mouse’s right ear. After 30 min, the mouse’s right ear was obviously red and swollen and the capillary congestion was severe. Cervical vertebrae were dislocated and sacrificed. The ears on both sides were quickly cut off, an 8 mm ear punch was used to punch holes at the same position on the ears on both sides and they were immediately weighed to calculate the weight difference [36].

The formula for the difference in ear weight is as follows:

(2) $$D=\left(W_{r}-W_{l}\right)$$

where $W_{r}$ was the weight of the right ear and $W_{l}$ was the weight of the left ear.

The formula for the inhibition rate of ear swelling is as follows:

(3) $$\mathrm{Inhibition}\;\left(\%\right)=\left[\frac{D_{c}-D_{t}}{D_{c}}\right]\times 100\%$$

where $D_{c}$ was the extent of ear edema of the control group and $D_{t}$ was the extent of ear edema of the treatment group.

### 3.15. Statistical Analysis

The data in this experiment were analyzed using SPSS (version 25.0, SPSS Inc., Chicago, IL, USA) software for one-way statistical analysis of variance, expressed as “Mean ± SD”. * *p* < 0.05, ** *p* < 0.01 indicates significance.

## 4. Conclusions

Five 1-methylhydantoin *trans*-cinnamic imides were prepared and their anti-inflammatory activities were evaluated. In the in vitro anti-inflammatory model established by LPS-stimulated RAW264.7 cells, compound **4** significantly inhibited the secretion of inflammatory factors TNF-α and IL-1β, and at the same time reduced the release of NO, showing good in vitro anti-inflammatory activity. In addition, in the in vivo anti-inflammatory model of xylene-induced ear swelling in mice, all five compounds (**1–5**) reduced the degree of ear edema and showed good anti-inflammatory activity in vivo. Compound **5** showed an obvious anti-inflammatory effect. Under the action of large dose, the inhibition rate of ear swelling was as high as 52.08%. In summary, these five target compounds can be used as new anti-inflammatory lead compounds, laying the foundation for their further development and utilization.

## Figures and Tables

**Table 1:** Purity data of 1-methylhydantoin cinnamic imides based on the Liquid Chromatographic Peak Area method.

Compound	Retention Time (min)	Plate Number	Purity (%)
**1**	3.586	10446	99.7
**2**	3.271	10189	99.0
**3**	3.397	9925	99.7
**4**	3.352	9201	95.5
**5**	3.368	9700	98.8

**Table 2:** The CC50 and safe concentration range of 1-methylhydantoin cinnamic imides on RAW264.7 cells.

Compound	R	CC50 (μM)	Concentration (μM)	Survival Rate (%)
**1**	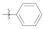	264	≤20	≥95
**2**	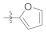	329	≤80	
**3**	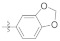	184	≤20	
**4**	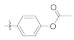	302	≤80	
**5**	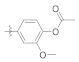	232	≤20	

**Table 3:** Docking scores of 1-methylhydantoin cinnamic imides to three target proteins.

Compound	COX-1 (kcal/mol)	COX-2 (kcal/mol)	iNOS (kcal/mol)	nNOS (kcal/mol)	eNOS (kcal/mol)	TNF-α (kcal/mol)	IL-1β (kcal/mol)
**MHD**	−4.8	−4.6	−4.8	−4.9	−4.5	−3.6	−4.4
**1**	−7.3	−7.3	−7.6	−8.6	−9.5	−5.6	−7.5
**2**	−6.9	−7.3	−8.2	−7.6	−7.9	−5.3	−6.8
**3**	−7.3	−7.5	−8.1	−8.1	−8.9	−6.1	−8.0
**4**	−6.9	−6.7	−7.1	−8.3	−8.8	−6.4	−7.5
**5**	−7.2	−6.7	−7.7	−8.2	−8.5	−6.3	−7.4

**Table 4:** Effect of 1-methylhydantoin cinnamic imides on xylene-induced ear edema in mice.

Group	Dose (mg/kg)	Degree of Edema (mg)	Inhibition (%)
Control	/	12.25 ± 1.48	/
**1**	100	6.84 ± 0.61 **	44.16
	50	8.47 ± 0.64 **	30.86
	25	9.84 ± 0.72 **	19.67
**2**	100	6.19 ± 1.13 **	49.47
	50	7.96 ± 0.23 **	35.02
	25	8.68 ± 0.79 **	29.14
**3**	100	8.20 ± 0.27 **	33.06
	50	9.05 ± 0.84 **	26.12
	25	9.41 ± 1.37 **	23.18
**4**	100	7.33 ± 0.51 **	40.16
	50	8.79 ± 0.76 **	28.24
	25	11.03 ± 0.32	9.96
**5**	100	5.87 ± 0.75 **	52.08
	50	7.95 ± 0.71 **	35.10
	25	10.46 ± 0.64 *	14.61

Values expressed as mean ± SD. (n = 6, * *p* < 0.05, ** *p* < 0.01 vs. control). Inhibition was expressed as a percentage.

## Data Availability

Not applicable.

## Supplementary Materials

The following supporting information can be downloaded at https://www.mdpi.com/article/10.3390/molecules27238481/s1, Figure S1: ^1^H- and ^13^C- NMR of compound **1**; Figure S2: ^1^H- and ^13^C- NMR of compound **2**; Figure S3: ^1^H- and ^13^C- NMR of compound **3**; Figure S4: ^1^H- and ^13^C- NMR of compound **4**; Figure S5: ^1^H- and ^13^C- NMR of compound **5**.

## Sample Availability

Samples of the compounds are not available from the authors.
