# Supplementary material for: Synthesis and Anti-Inflammatory Activity of 1-Methylhydantoin Cinnamoyl Imides
Source: Molecules. 2022 Dec 2;27(23):8481. doi: 10.3390/molecules27238481 (PMC9737772; doi:10.3390/molecules27238481)
Supplement: Supplementary file 1 [file molecules-27-08481-s001.zip › molecules-2017170-supplementary.pdf]

Synthesis and Anti-inflammatory Activity of 1-Methylhydantoin Cinnamoyl Imides

Shihan Wang,[1] Li Ji,[1] Dongxue Zhang,[1] Hongye Guo,[2] Yongsheng Wang[2] and Wei Li[1],*

[1]College of Chinese Medicinal Materials, Jilin Agricultural University, Changchun 130118, China

[2]School of Pharmaceutical Sciences, Jilin University, Changchun, Jilin 130021, China

*Correspondence: liwei7727@126.com

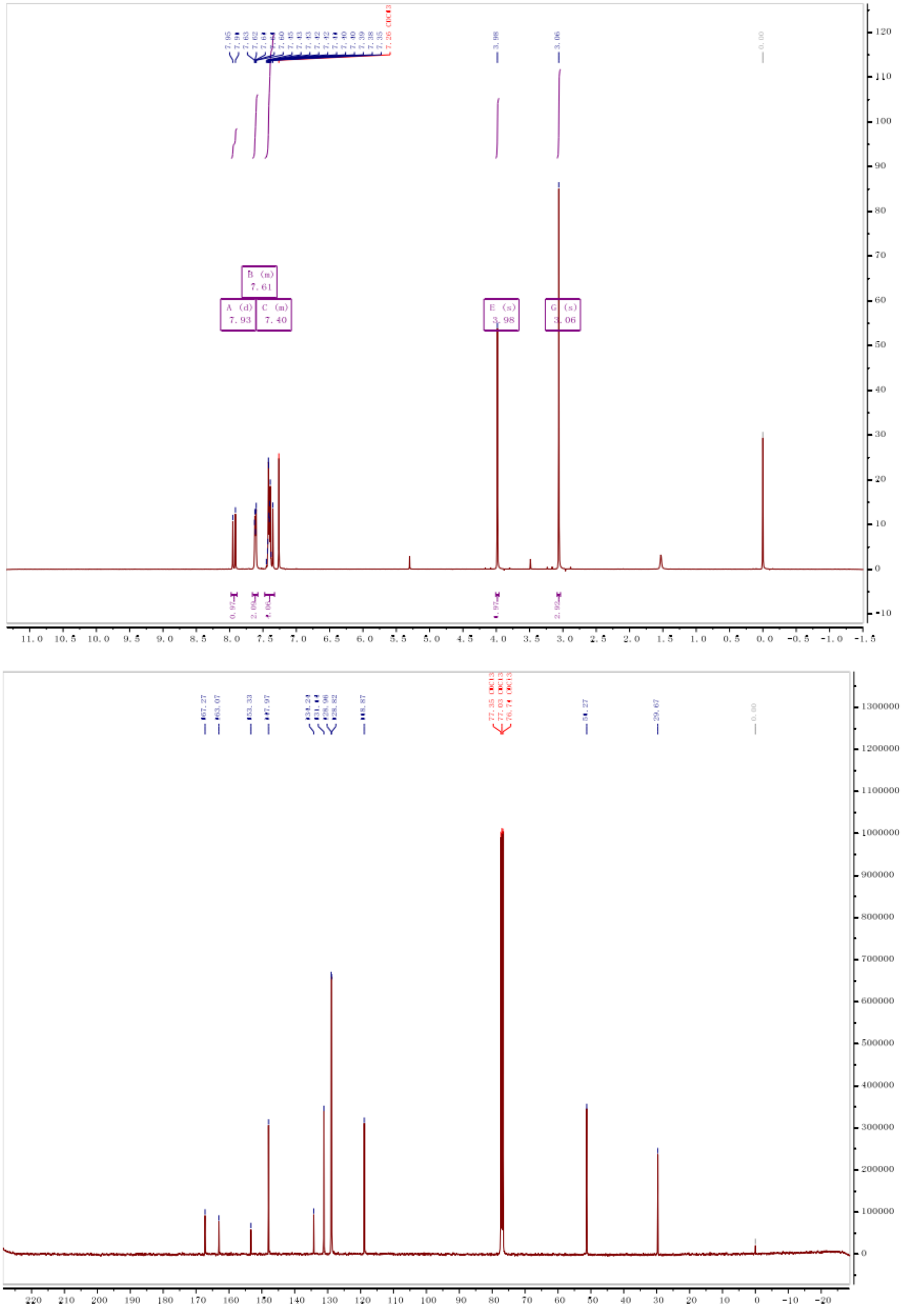

Figure S1 ^{1}H- and ^{13}C- NMR of compound **1**.

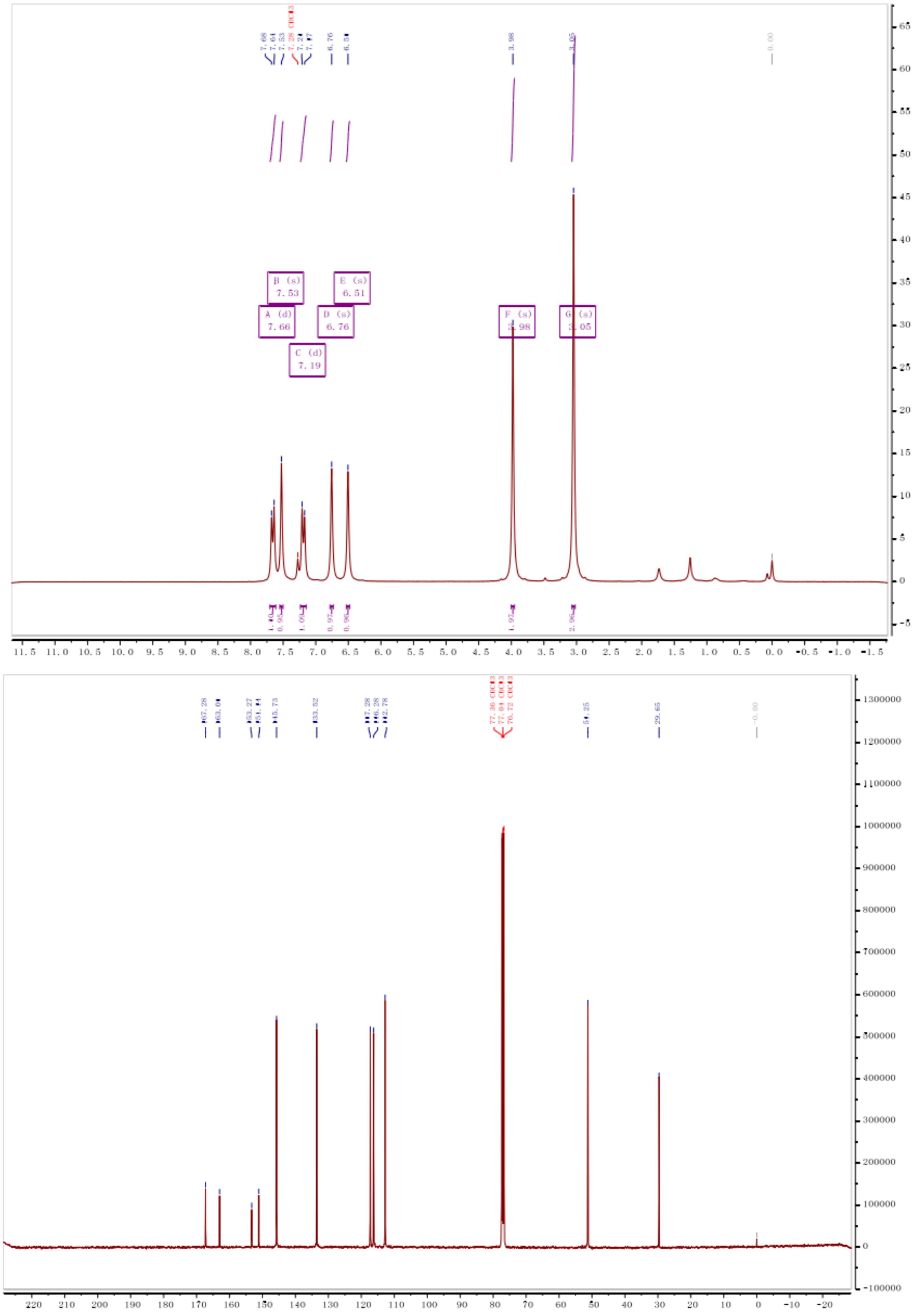

Figure S2 ^{1}H- and ^{13}C- NMR of compound **2**.

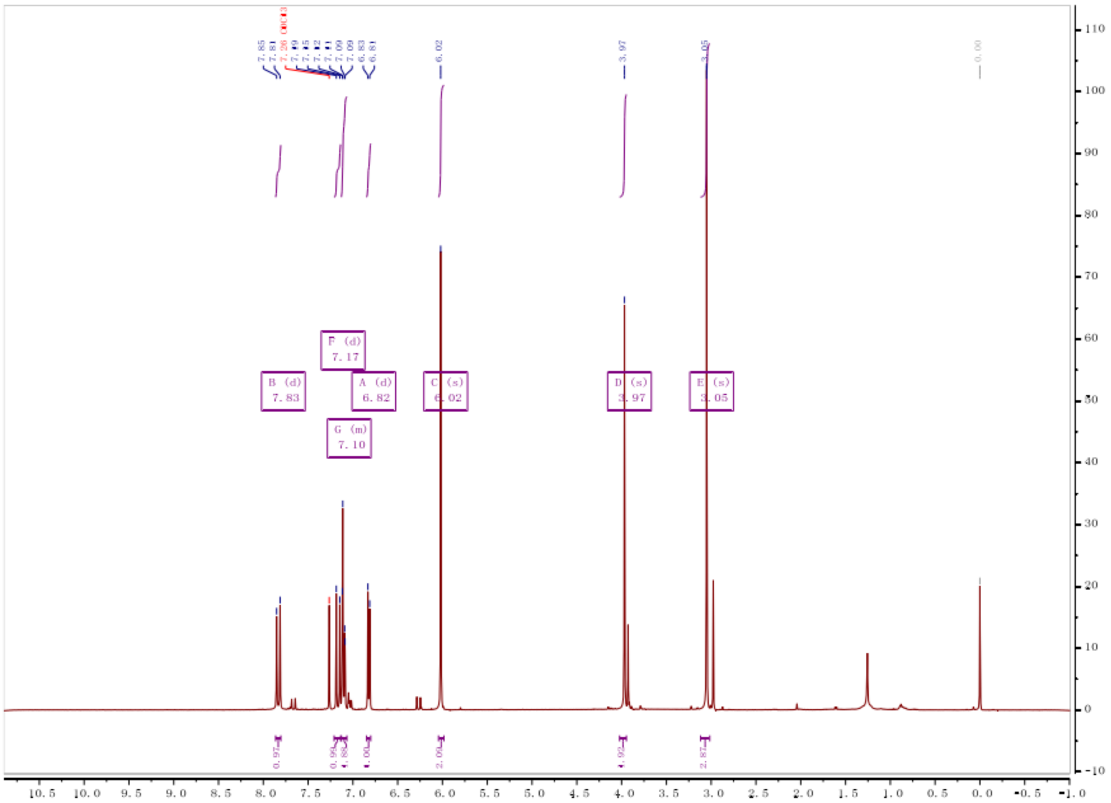

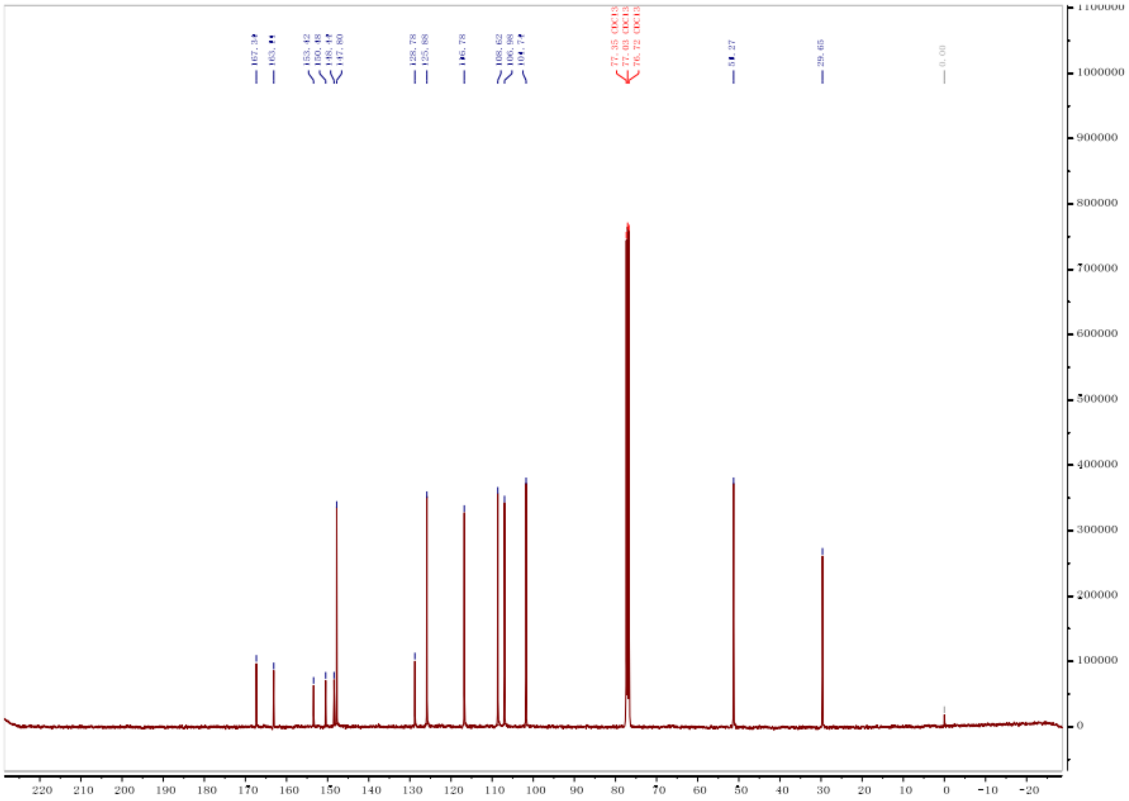

Figure S3 ^{1}H- and ^{13}C- NMR of compound **3**.

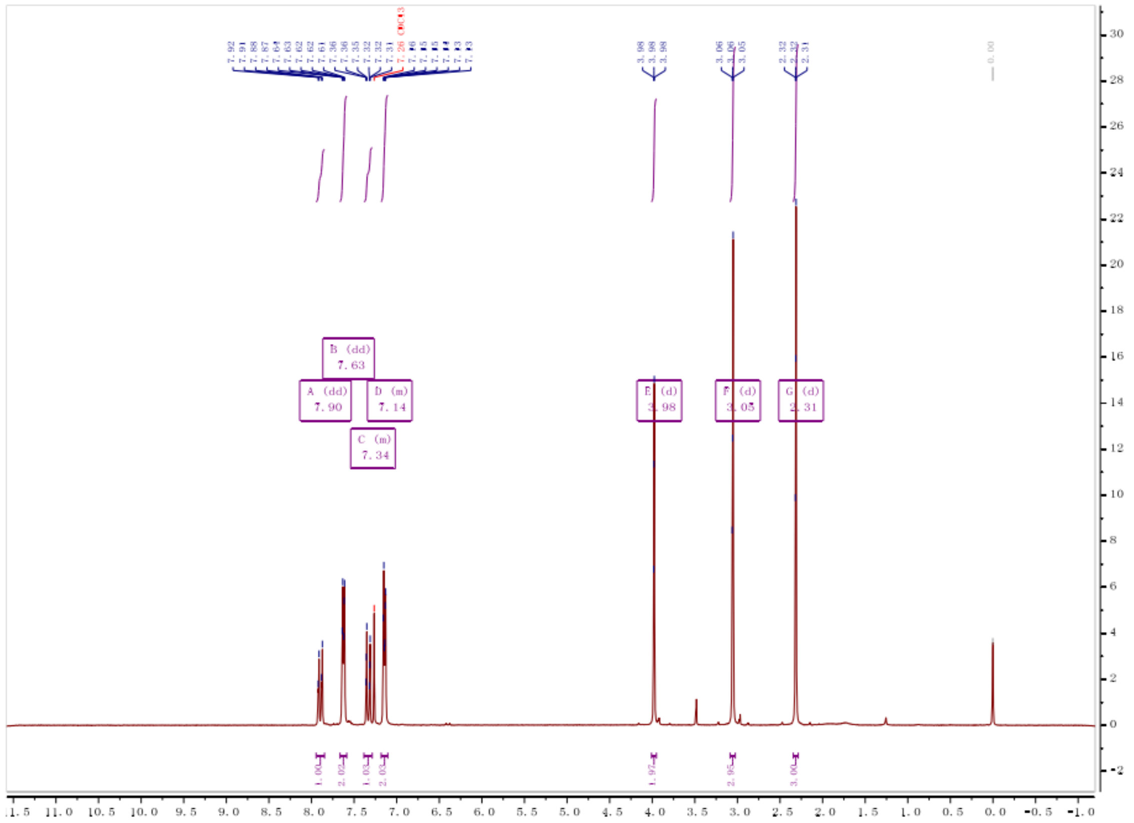

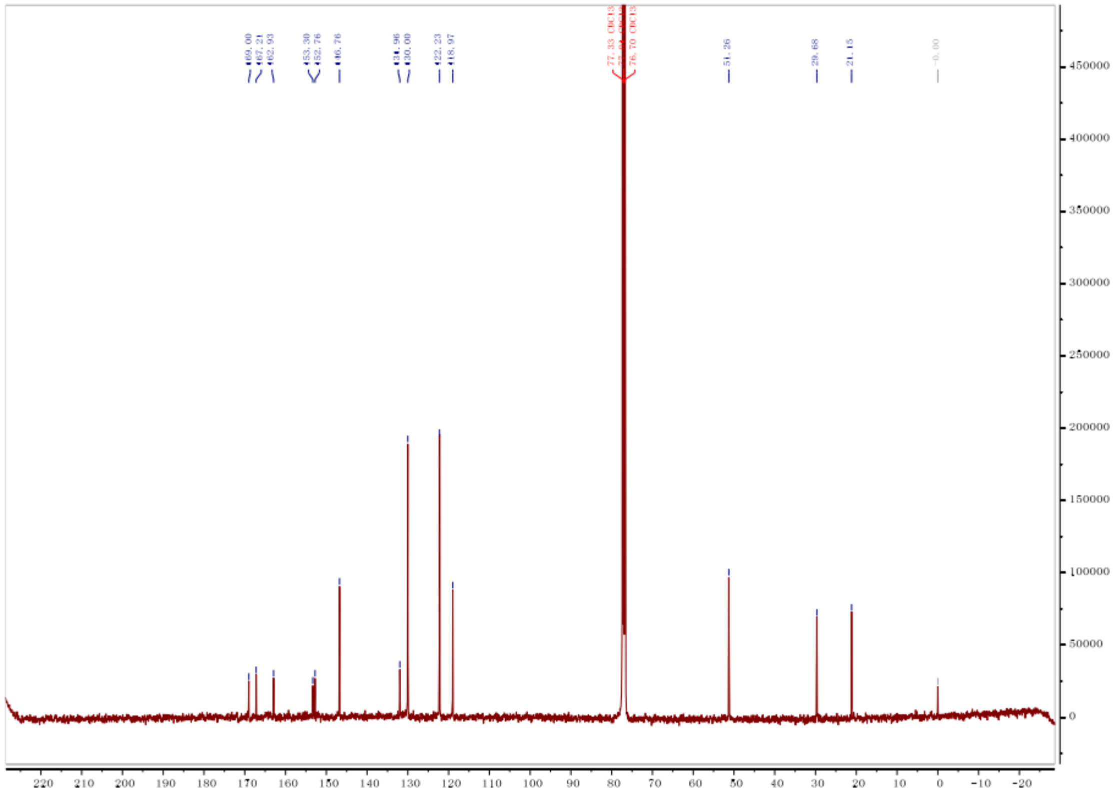

Figure S4 ^{1}H- and ^{13}C- NMR of compound **4**.

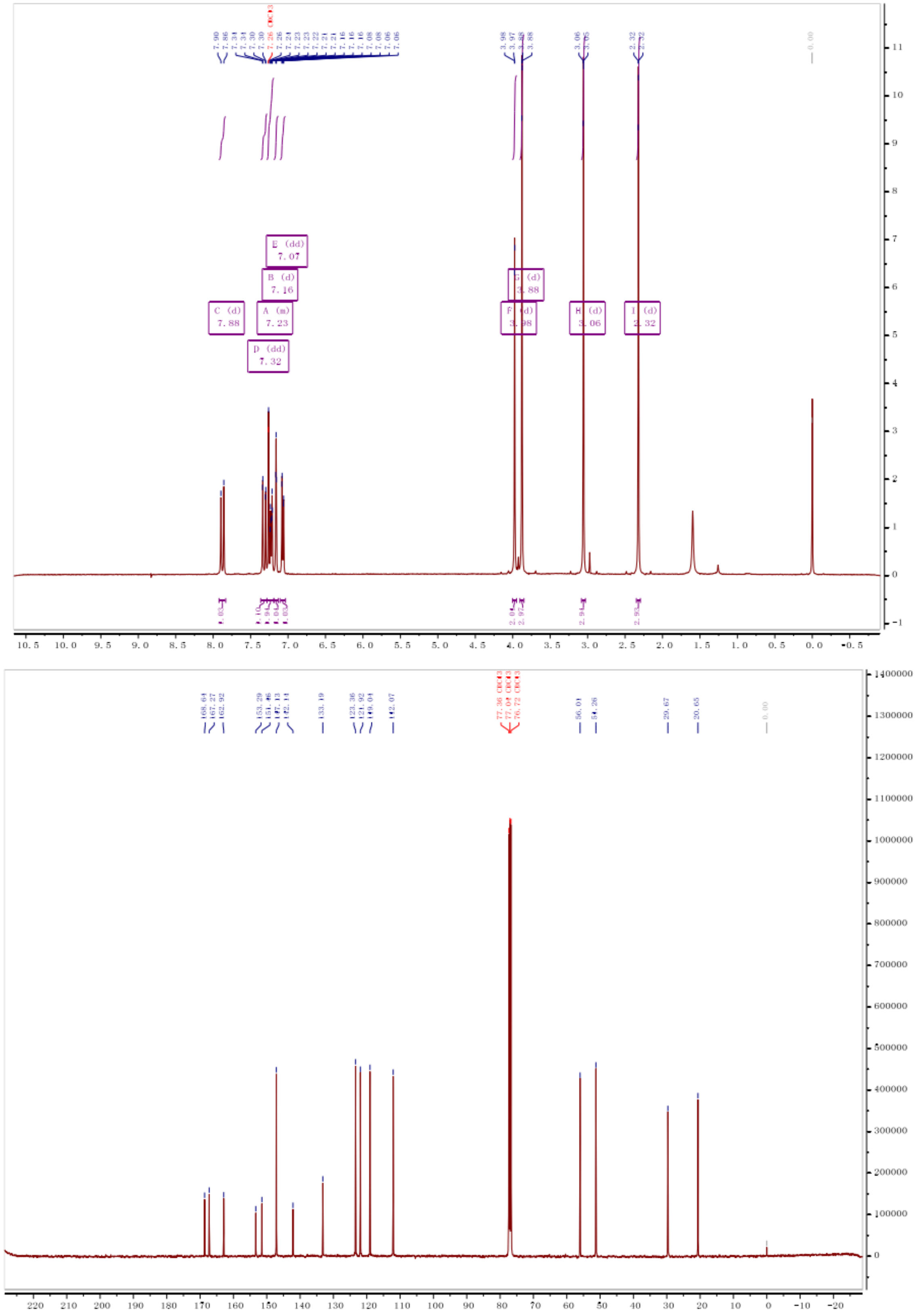

Figure S5 ^{1}H- and ^{13}C- NMR of compound **5**.
